# Comparison of Vision Transformers and Convolutional Neural Networks in Medical Image Analysis: A Systematic Review

**DOI:** 10.1007/s10916-024-02105-8

**Published:** 2024-09-12

**Authors:** Satoshi Takahashi, Yusuke Sakaguchi, Nobuji Kouno, Ken Takasawa, Kenichi Ishizu, Yu Akagi, Rina Aoyama, Naoki Teraya, Amina Bolatkan, Norio Shinkai, Hidenori Machino, Kazuma Kobayashi, Ken Asada, Masaaki Komatsu, Syuzo Kaneko, Masashi Sugiyama, Ryuji Hamamoto

**Affiliations:** 1 Division of Medical AI Research and Development, National Cancer Center Research Institute, 5-1-1 Tsukiji, Chuo-ku, Tokyo, 104-0045 Japan; 2 Cancer Translational Research Team, RIKEN Center for Advanced Intelligence Project, 1-4-1 Nihonbashi, Chuo-ku, Tokyo, 103-0027 Japan; 3 Department of Neurosurgery, Graduate School of Medicine, The University of Tokyo, 7-3-1 Hongo Bunkyo-ku, Tokyo, 113-8655 Japan; 4 Department of Surgery, Graduate School of Medicine, Kyoto University, Yoshida-konoe-cho, Sakyo-ku, Kyoto, 606-8303 Japan; 5 Department of Biomedical Informatics, Graduate School of Medicine, The University of Tokyo, 7-3-1 Hongo Bunkyo-ku, Tokyo, 113-8655 Japan; 6 Department of Obstetrics and Gynecology, Showa University School of Medicine, 1-5-8 Hatanodai, Shinagawa-ku, Tokyo, 142-8666 Japan; 7 RIKEN Center for Advanced Intelligence Project, Tokyo, 103-0027 Japan

**Keywords:** Artificial intelligence, Vision transformer, Convolutional neural network, Medical image analysis, Prior learning

## Abstract

In the rapidly evolving field of medical image analysis utilizing artificial intelligence (AI), the selection of appropriate computational models is critical for accurate diagnosis and patient care. This literature review provides a comprehensive comparison of vision transformers (ViTs) and convolutional neural networks (CNNs), the two leading techniques in the field of deep learning in medical imaging. We conducted a survey systematically. Particular attention was given to the robustness, computational efficiency, scalability, and accuracy of these models in handling complex medical datasets. The review incorporates findings from 36 studies and indicates a collective trend that transformer-based models, particularly ViTs, exhibit significant potential in diverse medical imaging tasks, showcasing superior performance when contrasted with conventional CNN models. Additionally, it is evident that pre-training is important for transformer applications. We expect this work to help researchers and practitioners select the most appropriate model for specific medical image analysis tasks, accounting for the current state of the art and future trends in the field.

## Introduction

**Fig. 1:**
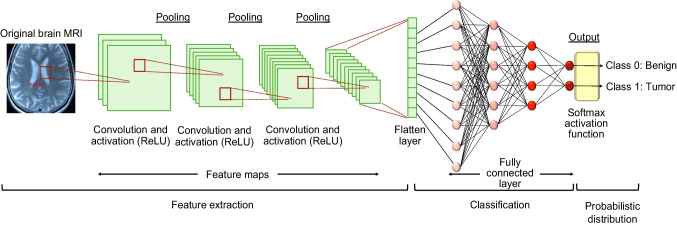
An example of medical image analysis using CNN (brain MRI). CNNs have input layers, output layers, many hidden layers, millions of parameters, and the ability to train complex objects and patterns. The input layer subsamples the input given by the convolution and pooling process and applies the activation function (ReLU in this figure). All of these layers are partially connected hidden layers, with the last fully connected layer being the output layer. The output retains its original shape, which is close to the dimensions of the input image

Convolutional neural networks (CNNs) are a type of deep learning algorithm and a key technology behind the modern field of artificial intelligence (AI) [1]. They consist of multiple convolutional layers with nonlinear activation functions, pooling layers, and fully connected layers, enabling them to capture complex patterns and textures in images, rendering them particularly well-suited for visual data interpretation applications [2]. Consequently, CNNs play a pivotal role in medical image analysis, extracting and improving the accuracy and efficiency of image-based medical applications. For instance, CNNs find application in the classification, segmentation, and registration of various medical images such as endoscopic, X-ray, magnetic resonance imaging (MRI), computed tomography (CT), ultrasound (US), skin, and histopathology images [3–10]. An example of the application of CNN to brain MRI is shown in Fig. 1. CNNs contribute to disease detection, including bone fractures, pneumonia, and cancer, as well as predicting cancer prognosis and pathological classification based on genetic mutations [11–16]. Moreover, a number of CNN-based segmentation models have been reported to exhibit performance comparable to that of human experts [17–19]. Despite the advanced capabilities of CNN in medical image analysis, they possess certain limitations. A primary concern is their lack of explainability; CNNs often operate as black boxes, providing minimal insights into how they reach conclusions, although several techniques, such as gradient-weighted class activation mapping (Grad-CAM), have been developed [20]. This opacity poses a significant challenge in clinical settings where understanding the decision-making process is crucial for diagnosis and treatment. Additionally, CNNs are prone to domain shift problems; for example, their performance may degrade when exposed to data differing from the training dataset, such as images from different medical centers or imaging devices [21–23]. This vulnerability raises concerns about the reliability and generalizability of these findings across different clinical environments.

**Fig. 2:**
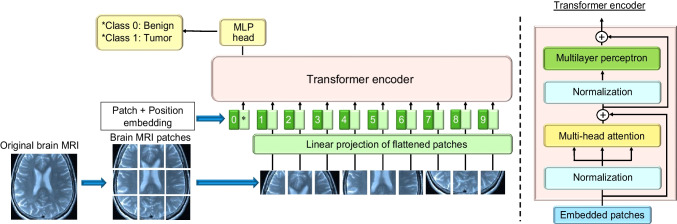
An example of medical image analysis using ViT (same brain MRI as used in Fig. 1). ViT consists mainly of the encoder part of the transformer. First, one input image is divided into *N* (*P*, *P*) resolution patches ($\:\mathcal{x}\in\:\:{\mathbb{R}}^{H\:\times\:W\:\times\:C\:}$→ $\:{\mathcal{x}}_{\varvec{p}}\in\:\:{\mathbb{R}}^{N\:\times\:({P}^{2}\bullet\:C)}$), where (*H*, *W*) is the resolution of the original image, and *C* is the number of channels. Then, with matrix **E**, project each patch onto a vector of length ($\:{P}^{2}\bullet\:C$) to *D* dimensions to create the location information of the original patch ($\:{\mathbf{E}}_{pos}\:\in\:\:{\mathbb{R}}^{(N+1)\:\times\:D}$). Combine these data as input data to the Transformer Encoder ($\:{z}_{0}=\:\left[{x}_{class};\:{x}_{p}^{1}\mathbf{E};\:{x}_{p}^{2}\mathbf{E};\:\bullet\:\bullet\:\bullet\:;\:{x}_{p}^{N}\mathbf{E}\right]\:+\:{\mathbf{E}}_{pos})$. The output of the Transformer-Encoder is further input to MLP to obtain task-specific output (Class 0: Benign, Class 1: Tumor, etc.)

Although other techniques have been employed, such as recurrent neural networks, convolution was the mainstream approach in the field of image processing [24] until very recently (in the 2020s). Transformers, originally developed for natural language processing (NLP), have revolutionized the field of deep learning due to their unique architectures based on self-attention mechanisms [25]. Vision transformers (ViTs) adopt this powerful framework for image processing [26]. An example is shown in Fig. 2. Unlike traditional convolutional approaches, ViTs treat an image as a sequence of patches and apply a transform model to these patches, enabling them to learn spatial hierarchies and relationships in the visual data [27, 28]. This approach has achieved remarkable success, offering an alternative to CNNs with potentially greater flexibility and the ability to handle more diverse and complex image datasets. ViTs and their derived instances achieved state-of-the-art (SOTA) performance on several benchmark datasets [29–31]. Active research has been conducted on adding explainability to ViTs, including the use of attention maps to visualize the features detected by ViTs [32–34]. Although ViTs have positive aspects, they face unique challenges. First, they require larger datasets for effective training compared to CNNs [35–37], which can be critical in situations with limited data, such as medical data. Second, ViTs often require additional computational resources, making their deployment challenging in resource-constrained environments [38–40]. Last, their relative novelty implies less established knowledge and best practices for their applications compared to those for CNNs.

While numerous new models based on convolution and attention mechanisms have been proposed, direct comparisons between these two architectures are relatively rare. This scarcity is likely due to the challenges involved in ensuring that learning conditions are strictly equivalent. Considering the above, our central research question is, “When building a machine learning model using medical images as input, which architecture should be used: CNNs or transformers?”

To accurately address this question, we pose several sub-questions:

- SQ1: Are there tasks that are well-suited for each model?
- SQ2: Are there appropriate image modality types for each model?
- SQ3: What are the optimal learning conditions for each model?
- SQ4: Which architecture demonstrates greater robustness?

**Fig. 3:**
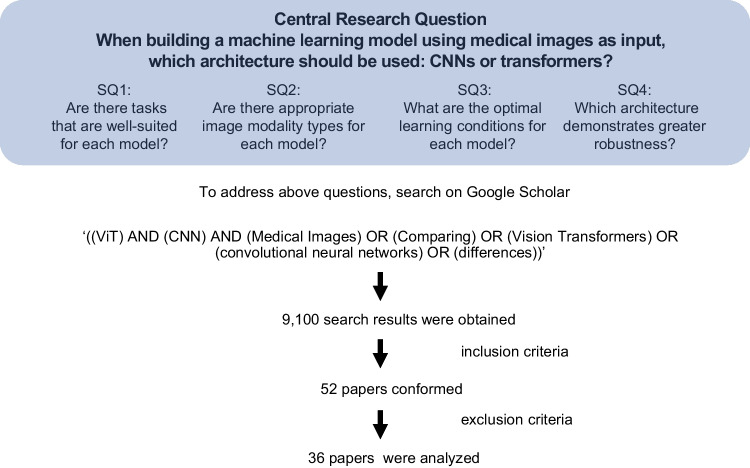
Overview of the central research question and sub-questions guiding the systematic review. The central research question, four sub-questions (SQ), search strategy, and number of articles retrieved and retained in analyses are indicated

To address these questions, we conducted a literature review. Figure 3 illustrates the central research question and sub-questions guiding our systematic review. The remainder of this paper is organized as follows: In the “Basic Concepts and Historical Overview”  section, we briefly present the basic concepts and historical overview of convolution and attention. The “Methods” section describes the research methodology, and the “Results” section presents the selected papers. Finally, in the “Discussion” section, we discuss the selected papers and answer the research questions.

## Basic Concepts and Historical Overview

### Concept of Convolution

In this section, we describe the concept of convolution in the context of CNNs. Convolution is a mathematical operation in which a filter comprising kernels is applied to an input image to extract its features. The convolution operation at position (*x*, *y*) is defined as:

1 $$\:\left(I\text{*}K\right)\left(x,y\right)={\sum\:}_{i=0}^{a}{\sum\:}_{j=0}^{b}I\left(x+i,y+j\right)\cdot\:K\left(i,j\right)$$

**Fig. 4:**
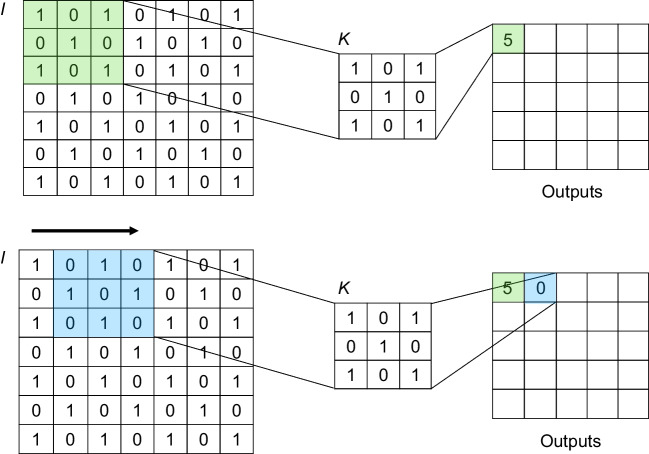
Illustration of the convolution operation. The input matrix *I* (left) is convolved with the kernel *K* (middle) to produce the output matrix (right). The convolution operation at position *(x*,* y)* is mathematically defined as:

*I* denotes the input image, and *K*, a 2-dimensional kernel of size *(a + 1*,* b + 1)*, represents the convolutional kernel; the convolution operation (denoted by ∗) at a position *(x*,* y)*, where *I (x + i*,* y + j)* denotes the pixel value of the image at position *(x + i*,* y + j);* and *K(i*,* j)* denotes the corresponding kernel value. Figure 4 illustrates the convolution operation. The kernel glides over the image, and the sum is calculated at each position to effectively filter the image. This process captures local patterns, such as edges, textures, and shapes, which are critical for image recognition tasks. Moreover, the depth, size, and number of kernels are key hyperparameters in CNNs that determine the ability of the network to extract different levels of features, from simple to complex. Learning in CNNs involves updating the kernel values, which is the essence of CNNs, allowing them to effectively learn hierarchies of image features.

$$\:\left(I\text{*}K\right)\left(x,y\right)={\sum\:}_{i=0}^{a}{\sum\:}_{j=0}^{b}I\left(x+i,y+j\right)\cdot\:K\left(i,j\right)$$

In this specific example, aaa and bbb are both equal to 2, as the kernel *K* is a 3 × 3 matrix (with indices ranging from 0 to 2). In the top part of the figure, the kernel *K* is positioned over the top-left corner of the input matrix *I*. Each element of the kernel is multiplied by the corresponding element of the input matrix, and the results are summed to produce a single value (5 in this case), which is placed in the top-left position of the output matrix. In the bottom part of the figure, the kernel *K* slides to the next position to the right on the input matrix *I*. The same element-wise multiplication and summation process is performed, resulting in a value of 0, which is placed in the corresponding position of the output matrix. This process is repeated across the entire input matrix to generate the full output matrix, capturing important features, such as edges and patterns, in the input data.

### Brief Historical Overview of Convolution

The concept of convolution in neural networks dates back to the 1980s with the introduction of the neocognitron by Kunihiko Fukushima in 1980 [41]. Inspired by the visual cortex of animals, this model included components called “simple” cells (S cells) and “complex” cells (C cells), laying the foundation for feature extraction through layered convolutions. In 1998, LeCun’s study marked an important milestone in the practical application of CNNs [42]. LeCun et al. demonstrated the effectiveness of CNNs in image-recognition tasks, particularly in the recognition of handwritten digits, using the LeNet-5 architecture. The popularity and utility of CNNs surged with the advent of deep learning and increased computing power in the 21st century [43]. The 2012 ImageNet Challenge (ISVRC-2012) was a pivotal moment where Krizhevsky’s AlexNet model significantly outperformed traditional image recognition methods [44]. This success showcased the power of deep CNNs in handling large-scale visual data and led to the rapid proliferation of CNN applications in various fields, particularly image and video recognition. Considerable progress has been made in the medical imaging applications of CNNs, including image anomaly detection, radiological image segmentation, and pathological slide analyses (Fig. 1) [7, 45–48]. The historical journey of CNNs, from their inception to their current status as the cornerstone of image analysis, highlights their transformative impact in both technology and healthcare fields.

### Advantages, Disadvantages, and Applications of Convolution

CNNs effectively capture local patterns, such as edges, textures, and shapes, within images. This local feature extraction is critical for tasks where detailed spatial hierarchies are important. A key advantage of CNNs is parameter sharing, where convolutional kernels are shared across different regions of the image [44]. This reduces the number of parameters, making the model less complex and easier to train. In addition, convolution operations can be efficiently parallelized, leading to faster computations on modern GPUs. However, CNNs have difficulty capturing long-range dependencies due to their localized receptive fields. Fixed kernel sizes limit the ability to detect features at different scales and in different contexts within the image. Despite these limitations, CNNs are widely used for image recognition tasks, such as object detection, face recognition, and image classification (e.g., the ResNet and VGG architectures). They are also widely used in medical image analysis, including the analysis of MRI and CT scans for disease detection and diagnosis, including the identification of tumors or anomalies.

### Concept of Attention

**Fig. 5:**
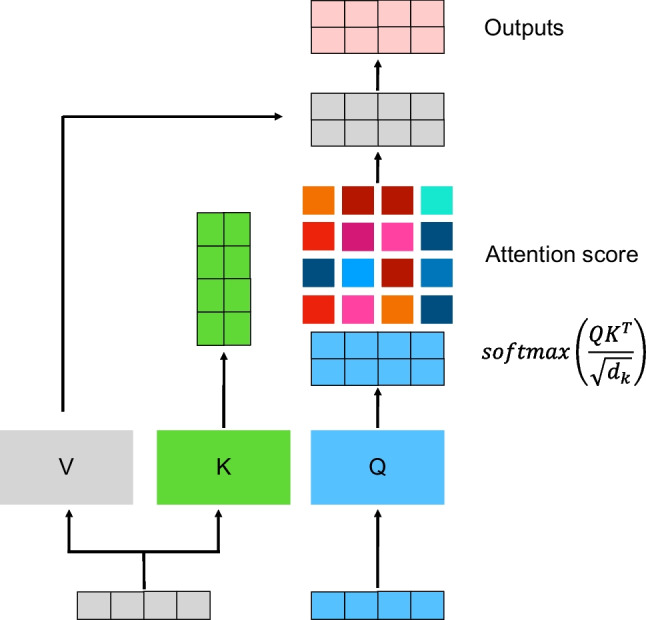
Illustration of the attention operation. The input data are split into three matrices: queries *(Q)*, keys *(K)*, and values *(V).* These matrices are generated from the input data through learned linear transformations

In deep learning, the attention mechanism, which is central to the structure of ViTs, signifies a paradigm shift from the localized receptive fields of CNNs. Vaswani et al. introduced attention in their seminal paper, “Attention is all you need.” The essence of the attention mechanism is to focus on different parts of the input data and dynamically weigh their importance [25]. The core concept can be encapsulated in the formula for the scaled dot product of attention as follows:

2 $$\:\text{Attention}\left(Q,K,V\right)=\text{softmax}\left(\frac{Q{K}^{T}}{\sqrt{{d}_{k}}}\right)V$$

where *Q*, *K*, and *V* denote the query, key, and value matrices, respectively, derived from the input data; *d*_*k*_ denotes the scaling factor; and *d*_*k*_ is the key dimension. Figure 5 illustrates the attention operation. The softmax function can be applied to the scaled dot products of queries with keys to provide a distribution of weights [49]. These weights can then be applied to the values, resulting in an output that reflects the focused areas of the input. This attention mechanism enables the model to consider the entire input sequence globally, making it particularly adept at capturing long-range dependencies in the data. In the context of medical imaging, ViTs can detect patterns and correlations across an entire image, potentially providing a more holistic and detailed understanding compared to that by the localized approach of CNNs.

1. *Query (Q*): Represents the set of vectors that will be compared against the key vectors to calculate attention scores.
2. *Key (K)*: Represents the set of vectors that the queries are compared to.
3. *Value (V)*: Represents the set of vectors that are weighted by the attention scores and combined to produce the output.

The attention scores were calculated using the scaled dot-product attention formula:

$$\:\text{Attention}\left(Q,K,V\right)=\text{softmax}\left(\frac{Q{K}^{T}}{\sqrt{{d}_{k}}}\right)V$$

where *d*_*k*_ ​ is the dimension of the key vectors. The softmax function was applied to the scaled dot products of the query and key vectors to obtain the attention weights, which indicate the importance of each value vector in producing the final output. These weights were then used to combine the value vectors, resulting in the output vectors. The final output matrix was obtained by applying these attention-weighted value vectors.

### Brief Historical Overview of Attention

The concept of attention in deep learning was initially introduced to address the limitations of sequential data processing, particularly in NLP. A seminal study marked the beginning of the evolution of attention mechanisms [50], demonstrating how attention allows a model to focus on different parts of the input sequence while generating each word of the output sequence, thereby improving the performance of machine translation systems. However, significant advancements occurred in 2017, as mentioned above [25]. This study introduced a transformer model that relies entirely on the attention mechanism and omits the recurrent layer commonly used in NLP tasks. The main concept introduced in this study was self-attention, which enables the importance of different parts of input data to be weighted relative to each other, thereby effectively capturing long-range dependencies. The introduction of transformers has marked a paradigm shift in deep learning. Originally designed for NLP, their architectures have proven highly effective for various tasks. The adaptability of the attention mechanism has led to its integration into various domains, including computer vision. The ViT model, introduced by Dosovitskiy et al. in 2020, adapts the transformer architecture to image classification tasks [26]. By treating an image as a sequence of patches and applying a self-attention mechanism, ViTs demonstrated remarkable performance, challenging the dominance of CNNs in image-related tasks. However, in medical imaging, the application of attention-based models is still in its infancy compared to that of CNNs. Nonetheless, early results are promising, particularly in tasks that require the analysis of large-scale patterns and contextual information in images (such as detecting anomalies in radiological scans or identifying patterns on histopathology slides) (Fig. 2) [49, 51, 52].

### Advantages, Disadvantages, and Applications of Attention

Attention mechanisms consider the entire input sequence, allowing them to effectively capture long-range dependencies and global context. As they can process entire sequences in parallel, they are efficient and scalable. They use a flexible weighting scheme, where each part of the input can be dynamically assigned different levels of importance, improving the focus on relevant features [25]. However, computing attention weights for all input pairs leads to higher computational complexity and resource requirements. These models also require large amounts of data for training and are prone to overfitting when applied to smaller datasets. Attention mechanisms are central to NLP tasks, such as machine translation, question answering, and document summarization, as demonstrated by models such as BERT and GPT. They are also used in image processing tasks, such as image generation and captioning; for example, ViTs and SWIN transformer have shown promising results in capturing complex visual patterns [53].

### Methods

The methodology utilized for conducting the literature review followed previously reported guidelines [54]. Google Scholar was used as the data source for extracting primary studies. The search strings used in the study were ‘((ViT) AND (CNN) AND (Medical Images) OR (Comparing) OR (Vision Transformers) OR (convolutional neural networks) OR (differences))’. The search was conducted in October 2023.

### Inclusion Criteria

The inclusion criteria for selecting papers were that they had to be written in English and published between January 2021 and October 2023. This timeframe was chosen because ViTs were not proposed until the end of 2020 [26]. In addition, the studies had to compare CNNs and ViTs on medical images using any pre-trained model of the two architectures. Studies proposing a hybrid architecture combining the two architectures into one, with their results compared, were also considered. The dataset used in the studies was not specific but had to be an image dataset suitable for classification using both deep learning architectures. Studies validated using externally independent datasets were preferred; however, those validated using a single dataset were also included.

### Exclusion Criteria

Studies exclusively focusing on one of the two deep learning architectures (i.e., ViTs or CNNs) were excluded. Another exclusion criterion was that papers with fewer than three citations were not considered.

## Results

### Search Results

**Fig. 6:**
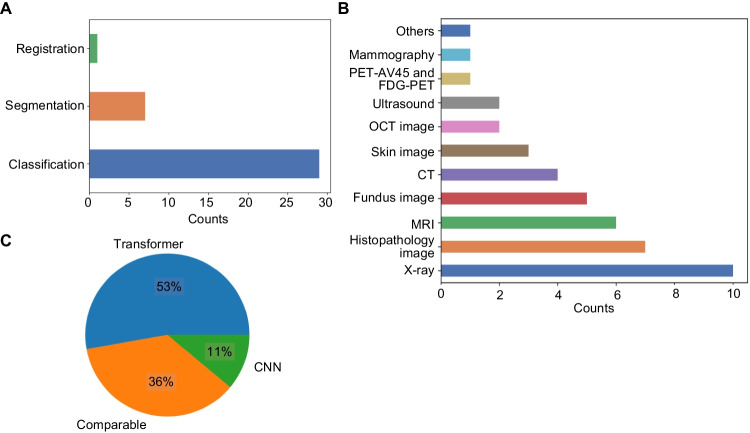
Summary of the 36 studies included in this study. (**A**) Distribution of task categories across 36 eligible studies. (**B**) Distribution of image modality types across 36 eligible studies. (**C**) The best technique determined by the study authors (subjective)

In this study, 9,100 search results were obtained using the search strings. Of these, 52 papers met the inclusion criteria, and 16 of these were excluded; accordingly, 36 papers were included in analyses (Fig. 3). While our initial criteria included a wide range of tasks, including detection, reconstruction, survival analysis and prediction, video-based applications, and image synthesis, no studies met our criteria within these specific tasks. An overview of the 36 included studies is presented in Tables 1 and 2. Figure 6A shows the distribution of task categories across 36 eligible studies. The most prevalent task was classification, followed by segmentation and registration. Figure 6B shows the distribution of image modalities across 36 eligible studies. Radiography was the most commonly used imaging modality, followed by pathological imaging, MRI, and fundus imaging. Figure 6C displays the results indicating which technique (convolution or attention) was deemed the most effective. It is important to note that these results were based on the authors’ descriptions and were therefore subjective. Additionally, validation with an independent external text dataset was performed in only two of the papers.

**Table 1:** Summary of 29 papers selected based on the search criteria for classification

Title	Task category	Task	Dataset	Image type	Independent external test dataset	Pre-training	Best architecture and its performance	Which is better?
A comparative evaluation between convolutional neural networks and vision transformers for COVID-19 detection [55]	Classification	COVID-19 or viral pneumonia or normal	COVID-QU-Ex	X-ray	No	Unknown	EfficientNetB7 (CNN-based) Best accuracy, 99.82%	Comparable
A comparative study between vision transformers and CNNs in digital pathology [56]	Classification	Tissue type identification, tumor detection	CRC9 and Camelyon16	Histopathology	No	ImageNet	Depending on datasets and evaluation criteria	Comparable
A Vision transformer for emphysema classification using CT images [57]	Classification	Emphysema subtype classification	COPD and private dataset	CT	Yes	ImageNet	ViT Best accuracy, 95.95%	Transformer
Advit: Vision transformer on multimodality PET images for Alzheimer’s disease diagnosis [58]	Classification	Alzheimer’s disease or not	ADNI	PET-AV45 and PET-FDG	No	ImageNet	Advit Best accuracy, 91%	Transformer
An improved transformer network for skin cancer classification [59]	Classification	Skin cancer classification	HAM10000 and private dataset	Skin	No	Unknown (for private dataset pretrained on HAM10000 dataset)	Proposed ViT model Best accuracies, 94.3% and 94.1% (HAM10000, private dataset)	Transformer
Analyzing transfer learning of vision transformers for interpreting chest radiographs [60]	Classification	14 pathologies (CheXpert), pneumonia or not	CheXpert and pediatric pneumonia dataset	X-ray	No	ImageNet	ViT Best accuracy, 87% (Pediatric pneumonia dataset)	Transformer
Convolution neural networks and self-attention learners for Alzheimer dementia diagnosis from brain MRI [61]	Classification	Alzheimer’s disease or normal controls	ADNI and OASIS	MRI	No	Unknown (CNN-based models), ImageNet (Transformer-based models)	DeiT Best accuracies, 75.625% and 72.562% (ADNI, OASIS dataset)	Transformer
CoViT-GAN: Vision transformer for COVID-19 detection in CT scan images with self-attention GAN for data augmentation [62]	Classification	COVID-19 or not	COVID-CT and SARS-CoV-2	CT	No	ImageNet	CoViT-GAN (ViT trained on augmentation images made by GAN) Best accuracies, 87.19% and 95.41% (COVID-CT, SARS-CoV-2 datasets)	Transformer
Delving into masked autoencoders for multilabel thorax disease classification [63]	Classification	Multiclass thorax disease classification (i.e., Nodule, pneumothorax)	NIH Chest X-ray 14 (75, 312 X-rays), Stanford CheXpert (191, 028 X-rays), and MIMIC-CXR (243, 334 X-rays)	X-ray	No	ImageNet	ViT Best accuracy, 91% (CheXpert)	Comparable
Detecting pneumonia using vision transformer and comparison with other techniques [64]	Classification	Pneumonic or normal	Public dataset	X-ray	No	ImageNet (VGG16), unknown (ViT)	ViT Best accuracy, 86.38%	Comparable
Detecting tuberculosis-consistent findings in lateral chest X-radiographs using an ensemble of CNNs and vision transformers [65]	Classification	Tuberculosis or not	Detecting tuberculosis	X-ray	No	ImageNet	DenseNet-121 Best accuracy, 85.85%	CNN
Diabetic retinopathy detection using CNN-, transformer- and MLP-based architectures [66]	Classification	Five categories classification: no diabetic retinopathy (DR), mild DR, moderate DR, severe DR, and proliferative DR	APTOS	Fundus	No	Unknown	Swin Transformer Best accuracy, 86.4%	Transformer
Explainable vision transformers and radiomics for COVID-19 detection in chest radiographs [33]	Classification	COVID-19 or viral pneumonia or normal	SIIM-FISABIO-RSNA COVID-19	X-ray	No	Unknown	ViT-B32 Best accuracy, 96%	Transformer
Focused attention on transformers for interpretable classification of retinal images [67]	Classification	OCT: four classes: Normal, Drusen, choroidal neo-vascularization (CNV) and diabetic macular edema (DME)., Fundus: the severity of DR (none, mild, moderate, severe, and proliferative)	OCT: UCSD OCT, HMR AROI Fundus: EyePACS, Aptos, and IDRiD	Fundus and OCT	Yes	ImageNet	Depending on datasets and task	Comparable
IEViT: An enhanced vision transformer architecture for chest X-ray image classification [68]	Classification	Depending on the dataset (i.e., Normal or COVID)	Kermany et al. dataset [69], Tuberculosis Chest X-ray, COVID-19 radiography, and COVIDx	X-ray	No	ImageNet	IEViT-L/32 (Proposed model) Best accuracy, 98.08% (Pediatric pneumonia dataset)	Transformer
Identifying malignant breast ultrasound images using ViT-patch [70]	Classification	Benign or malignant	The breast ultrasound dataset is from Al-Dhabyani et al. [71].	Ultrasound	No	Unknown	ViT/ViT-Patch Best accuracies, 85.6% and 89.0% (ViT/ViT-Patch)	Comparable
Image transformers for classifying acute lymphoblastic leukemia [72]	Classification	Normal or malignant cell	B-ALL blood cancer (C-NMC)	Cell image	No	Unknown	ViT Best accuracy, 88.4%	Comparable
Investigating vision transformer models for low-resolution medical image recognition [73]	Classification	Specific classifications	DermaMNIST, BloodMNIST, PneumoniaMNIST, and OrganCMNIST	Low-resolution medical image	No	Unknown	CNN Best accuracies, 74%, 93%, 88%, 86% (Derma, Blood, Pneumonia, OrganC)	CNN
Method for diagnosis of acute lymphoblastic leukemia based on ViT-CNN ensemble model [74]	Classification	Cancer or normal cells	ISBI 2019	Cell image	No	Unknown	ViT-CNN ensemble model Best accuracy, 99.03% (Pediatric pneumonia dataset)	Transformer
On the effectiveness of 3D vision transformers for the prediction of prostate cancer aggressiveness [75]	Classification	Low grade or High grade	ProstateX-2	MRI	No	No (Scratch)	2D-CNN Best accuracy, 73.8%	CNN
Pretrained ViTs yield versatile representations for medical images [76]	Classification and segmentation	Classification and segmentation	Classification: APTOS 2019, CBIS-DDSM, ISIC 2019, CheXpert, PatchCamelyon, and ISIC 2018	Skin, Fundus, Histopathology, X-ray, and Mammography	No	Random or ImageNet	Depending on datasets	Transformer
Transfer learning for histopathology images: an empirical study [77]	Classification	Lung images: three class classification (adenocarcinoma, lung squamous cell carcinoma, and benign lung tissue), colon images: two class classification (colon adenocarcinoma and benign colon tissue)	LC25000	Histopathology	No	ImageNet	ViT-L32 ensemble model Best accuracy, 99.77%	Comparable
ViT-DR: Vision transformers in diabetic retinopathy grading using fundus images [78]	Classification	Five-category classification: no diabetic retinopathy (DR), mild DR, moderate DR, severe DR, and proliferative DR	Kaggle and IDRiD	Fundus	No	Unknown	ViT Best accuracy, 87.41%	Transformer
ViT-P: Classification of genitourinary syndrome of menopause from OCT images based on vision transformer models [79]	Classification	Classification of genitourinary syndrome (normal, GSM, and UT)	GSM	OCT	No	Unknown	ViT-P (Proposed hybrid model) Best accuracy, 99.9%	CNN
Vision transformer-based recognition of diabetic retinopathy grade [80]	Classification	Diabetic retinopathy detection	Kaggle diabetic retinopathy detection dataset	Fundus	No	ImageNet	ViT Best accuracy, 91.4%	Transformer
Vision transformer for femur fracture classification [81]	Classification	AO/OTA proximal femur classification	Private dataset	X-ray	No	No (Scratch)	ViT Best accuracy, 83%	Transformer
Vision transformer for classification of breast ultrasound images [82]	Classification	Benign, malignant, or normal	BUSI and B dataset	Ultrasound	No	ImageNet	ViT-B32 Best accuracy, 86.7%	Comparable
Visual Transformers and convolutional neural networks for disease classification on radiographs: a comparison of performance, sample efficiency, and hidden stratification [83]	Classification	Two tasks: (a) diagnosis of thoracic diseases on chest radiographs and (b) diagnosis of abnormalities on upper extremity radiographs	(a) chest X-ray 14, CheXpert, PadChest, and MIMIC (b) MURA	X-ray	Yes	ImageNet	DenseNet-121 Best weighted area under the curve, 0.79	Comparable
Is the aspect ratio of cells important in deep learning? A robust comparison of deep learning methods for multiscale cytopathology cell image classification: From convolutional neural networks to visual transformers [84]	Classification	Two tasks: (a) dyskeratotic, koilocytotic, metaplastic, parabasal, and superficial intermediate, (b) abnormal or normal	(a) SIPaKMeD (b) HErlev	Cytopathology cell image	No	ImageNet	Depending on datasets and task	Comparable

**Table 2:** Summary of seven papers selected based on the search criteria for registration and segmentation

Title	Task category	Task	Dataset	Image type	Independent external test dataset	Pre-training	Best architecture and its performance	Which is better?
Affine medical image registration with coarse-to-fine vision transformer [85]	Registration	Two tasks: brain template-matching normalization to MNI152 space and Atlas-based registration in native space	OASIS and LPBA	MRI	No	Unknown	C2FViT (Proposed model) Best dice similarity coefficient, 0.76 ± 0.05 (MINI152)	Comparable
Convolution-free medical image segmentation using transformers [86]	Segmentation	Atlas segmentation	Brain cortical plate, pancreas, and hippocampus dataset	MRI, CT	No	Unknown	Proposed transformer model Best dice similarity coefficient, 0.879 ± 0.0526 (brain cortical plate)	Transformer
Evaluating transformer-based semantic segmentation networks for pathological image segmentation [87]	Segmentation	Tumor area segmentation for pathological image	PAIP grand challenge dataset	Histopathology	No	Unknown (Segmenter, Swin-Transformer, and TransUNet were trained on ImageNet)	Segmenter Best average Jaccard index, 0.82 ± 0.11 (brain cortical plate)	Transformer
Medical image segmentation using transformer networks [88]	Segmentation	Atlas segmentation	Public and private dataset	MRI	No	Unlabeled dataset (dHCP and public CT dataset)	Proposed networks Best dice similarity coefficient, 0.878 ± 0.037 (brain cortical plate)	Transformer
Skin lesion segmentation based on vision transformers and convolutional neural networks-a comparative study [89]	Segmentation	Skin lesion segmentation	ISIC 2018	Skin	No	Unknown	TransUNet Best dice similarity coefficient, 0.8984	Comparable
Swin UNETR: Swin transformers for semantic segmentation of brain tumors in MRI images [90]	Segmentation	Brain tumor segmentation	BraTS 2021	MRI	No	Unknown	Swin UNETR Best average dice similarity coefficient, 0.913	Transformer
Swin-Unet: Unet-like pure transformer for medical image segmentation [91]	Segmentation	Multiorgan segmentation	Synapse multiorgan segmentation dataset	CT	No	ImageNet	Swin-Unet Best dice similarity coefficient, 0.7913	Transformer

### Classification

Classification emerged as one of the most researched topics, with X-ray imaging being the most commonly used imaging type; hence, this combination was the most common, featured in ten papers. The popularity of X-ray imaging can be attributed to the ready availability of X-ray imaging data and numerous public X-ray imaging datasets. For instance, Nafisah et al., Usman et al., Tyagi et al., Chetoui et al., Okolo et al., and Murphy et al. attempted to detect pneumonia [33, 55, 60, 64, 68, 83]. Among these studies, the most noteworthy is that of Murphy et al. who utilized an independent external text dataset to rigorously compare the two architectures [83]. This study evaluated the target model performance, sampling efficiency, and hidden layer stratification in the analysis of chest and extremity radiographs. The key findings indicated that while CNNs, especially DenseNet121, slightly outperformed the data-efficient image transformer (DeiT)-B ViT in terms of diagnostic accuracy, both models demonstrated comparable sample efficiencies. Considerably, the ViT model exhibited reduced susceptibility to hidden stratification, a phenomenon in machine learning models in which predictions are made based on features not directly related to the condition or disease being diagnosed but rather on incidental, non-disease features present in the data. For example, in the context of disease classification using X-ray imaging, a model might erroneously associate the presence of a medical device, such as a chest tube, with a particular disease, such as pneumothorax. This misunderstanding is not because the medical device is an actual indicator of the disease but because the model has learned to correlate the presence of the device with the disease due to biases in the training data. Murphy et al. reported that the ViT model exhibited a lower tendency for hidden stratification than did the CNN model, suggesting that ViT is less susceptible to being misled by incidental features in medical images, potentially leading to more accurate and reliable disease classification.

Wu et al. conducted a study using an independent test dataset [57]. These findings demonstrate the potential of ViT in medical imaging, specifically for classifying emphysema subtypes. This study was notable for its ability to classify centrilobular, panlobular, and paraseptal emphysema from CT images, outperforming CNNs such as AlexNet, Inception-V3, and ResNet50 in terms of accuracy. A key aspect of this study was the use of a private dataset for training and a public dataset for testing. The ViT model achieved average accuracies of 95.95% and 72.14% for the private and public datasets, respectively, thus outperforming each CNN model. Moreover, the results were particularly good for the private test data and public test data, achieving average accuracies of 72.14% (ViT) vs. 66.07% (AlexNet, the best performance among the CNNs). This research highlighted the efficiency of ViT in handling data, requiring fewer images for training than do CNN models. Additionally, the study examined the interpretability of the model using attention rollout heat maps to visualize how ViT discriminated between different lung regions to classify emphysema types.

Fundus images are a relatively popular image type. Using the Kaggle diabetic retinopathy detection dataset [92], which poses challenges due to class imbalances, Wu et al. used data augmentation techniques (such as panning and rotating images) to enhance model training [80]. The methodology involved dividing fundus images into non-overlapping patches, linearly and positionally embedding them, and processing them through multihead attention layers in the ViT model. This approach yielded excellent results, with the model achieving an accuracy of 91.4%, specificity of 97.7%, precision of 92.8%, sensitivity of 92.6%, quadratic weighted kappa score of 0.935, and area under the curve (AUC) of 0.986, outperforming CNN models.

Deininger et al. compared the effectiveness of ViTs and CNNs in the field of digital pathology [56]. This study focused on the application of ViTs for tumor detection and tissue-type identification in whole-slide images (WSIs) of four different tissue types. The patchwise classification performance of the ViT model DeiT-Tiny was compared with that of the state-of-the-art (SOTA) CNN model ResNet18. Due to the limited availability of annotated WSIs, both models were trained on large volumes of unlabeled WSIs using self-supervised methods. The results showed that the ViT model slightly outperformed the ResNet18 model in tumor detection for three of the four tissue types, while the ResNet18 model performed slightly better in the remaining tasks. The aggregated predictions of both models correlated at the slide level, suggesting that they captured similar image features. Overall, the ViT model performed comparably to the ResNet18 model but required more training effort.

Pachetti et al. conducted a study using MRI-based images [75]. This study introduced and evaluated a modified 3D ViT architecture trained from scratch on the ProstateX-2 Challenge dataset. This study aimed to determine whether 3D ViTs could effectively predict the aggressiveness of prostate cancer based on the Gleason score, which has been diagnosed in a more invasive way. A key aspect of this research was a comparison of the performance of 3D ViT against a 3D CNN trained from scratch. The results showed that 3D ViT not only had the ability to predict cancer aggressiveness but also outperformed the 3D CNN in this task. However, the SOTA 2D CNN model—that is, the fine-tuned AlexNet model—outperformed the 3D ViT.

Gheflati and Rivaz presented a study on the use of ViT and CNN for breast US image classification [82]. Using two datasets with 943 breast US images, different pre-trained ViT models were compared with SOTA CNNs (ResNet50, VGG16, and NASNET models). A key finding was that ViTs, particularly the B/32 model, achieved high classification accuracy and AUC values that surpassed or matched those of the best CNN models. For example, the B/32 model achieved an accuracy of 86.7% and AUC of 0.95, demonstrating the potential of ViTs to efficiently process spatial information in medical images. An important aspect of this study was the demonstration that ViTs could achieve high performance even with smaller datasets, which is a considerable advantage in medical imaging, where large datasets are not always available.

The SkinTrans model proposed by Xin et al. was designed to focus on the most important features of skin cancer images while minimizing noise through a combination of multi-scale image processing and contrastive learning [59]. Two datasets were used for validation: the publicly available HAM10000 dataset, comprising 10,015 images from seven skin cancer classes, and a clinical dataset collected through dermoscopy, comprising 1,016 images, including three typical types of skin cancer. The SkinTrans model exhibited impressive results, achieving 94.3% accuracy on the HAM10000 dataset and 94.1% accuracy on the clinical dataset. The addition of the simple ViT model also achieved high accuracies of 93.5% and 93.4% on the respective datasets, outperforming CNN models. A notable aspect of this study was the use of Grad-CAM visual analysis, demonstrating that the proposed model could identify the most relevant areas in skin cancer images, indicating that it learned the correct features for accurate classification.

### Segmentation

**Fig. 7:**
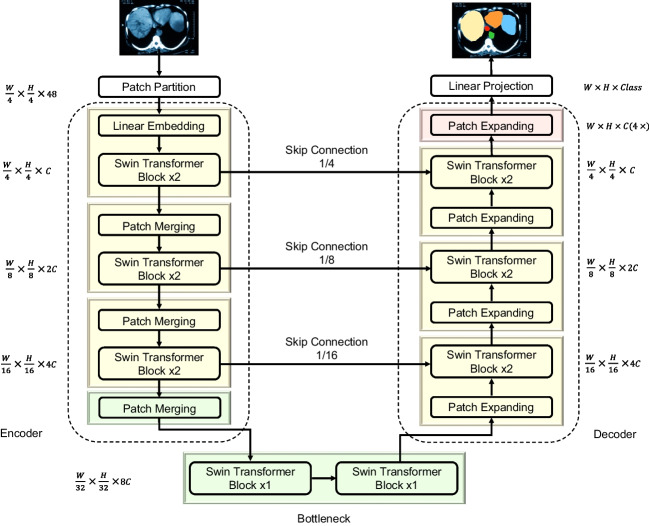
Example of medical image segmentation using Swin-Unet (lung CT image). The architecture was adapted from Cao et al. [91]. Swin-Unet consists of encoder, bottleneck, decoder, and skip connections. In the encoder, to convert the input to sequence embedding, a Linear Embedding layer is applied to project the feature dimension into an arbitrary dimension (represented as *C*) after the lung CT image is divided into 4 × 4 sized patches. The transformed patch token passes through several Swin Transformer Blocks and Patch Merging layers to produce a hierarchical feature representation. The decoder consists of Swin Transformer Blocks and Patch Expanding Layers. The extracted context features are fused with the multiscale features from the encoder via skip connections to complement the loss of spatial information due to down-sampling. The Patch Expanding layer reshapes the feature maps in adjacent dimensions into a larger feature map with twice the resolution up-sampled. The last Patch Expanding layer is used to perform 4 x up-sampling to restore the feature map resolution to the input resolution (*W* x *H*), and then a linear projection layer is applied to these up-sampled features to output pixel-level segmentation predictions

**Fig. 8:**
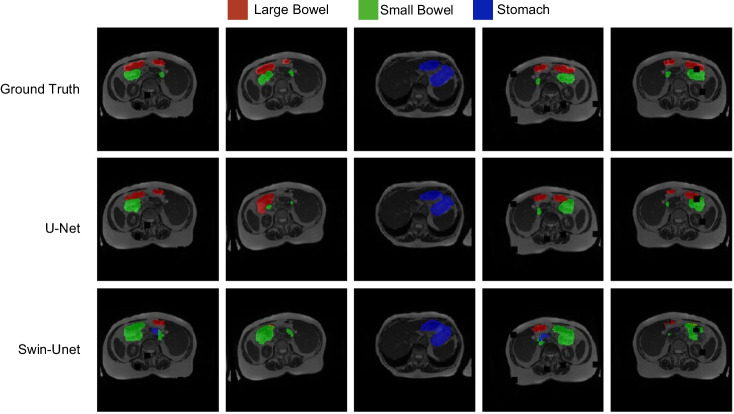
Comparison of segmentation results using U-Net and Swin-Unet on the UW-Madison GI Tract Image Segmentation dataset. The first row shows the ground truth annotations for the large bowel (red), small bowel (green), and stomach (blue). The second row presents the segmentation results from U-Net, trained for 100 epochs using the Adam optimizer with a learning rate of 2e-3, weight decay of 1e-6, and CosineAnnealingLR scheduler. The third row shows the segmentation results from Swin-Unet, trained for 100 epochs using the SGD optimizer with a learning rate of 0.05, momentum of 0.9, weight decay of 1e-4, and PolynomialDecayLR scheduler

Segmentation was the second most popular task and is expected to have many practical applications. Swin-Unet, proposed by Cao et al. is a new purely transducer-based segmentation model (Fig. 7) [91]. For example, Swin-Unet is characterized by using non-overlapping image patches as tokens, processed through a transformer-based encoder-decoder structure with skip links. This design facilitates effective learning of local and global semantic features. Studies have demonstrated the superior performance of Swin-Unet in segmentation tasks on multiple datasets, including multiorgan and cardiac segmentations, highlighting its excellent accuracy [91]. Figure 8 shows an illustrative example of segmentation predictions by U-Net and Swin-Unet, both trained on the UW-Madison GI Tract Image Segmentation dataset, using the validation portion of the same dataset for reference [93, 94]. Hatamizadeh et al. proposed the Swin-UNEt TRansformer (Swin UNETR), which uses Swin transformers as encoders in a U-shaped network connected to a multiresolution CNN-based decoder via skip links [90]. Swin UNETR is characterized by its ability to learn multi-scale contextual information and model long-range dependencies, outperforming previous methods in Brain Tumor Segmentation (BraTS) 2021, a brain tumor segmentation challenge [95].

Gulzar and Khan presented an in-depth comparison of ViTs and CNNs for skin lesion segmentation in medical images [89]. This research is crucial for evaluating the effectiveness of these technologies in medical image analysis, particularly in the challenging areas of skin lesion detection and segmentation. Using the ISIC 2018 dataset [96], different architectures, including the U-Net, V-Net, Attention U-Net, TransUNet, and Swin-Unet models, were examined, and their performance in accurately segmenting skin lesions was evaluated. The results highlighted that the hybrid models, particularly TransUNet, exhibited superior performance in terms of accuracy and the Dice coefficient, outperforming other benchmarking methods. This study highlighted the potential benefits of integrating ViTs with traditional CNNs in medical imaging and demonstrated their effectiveness in handling complex tasks, such as skin lesion segmentation.

### Registration

**Fig. 9:**
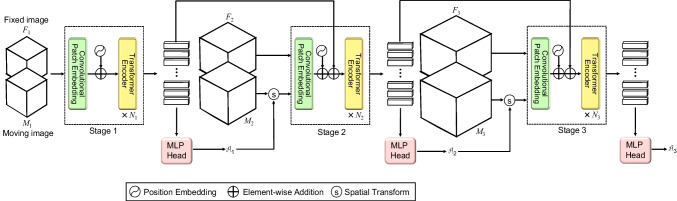
Overview diagram of C2FViT. This figure was prepared with reference to Mok et al. [85]. C2FViT uses convolutional patch embedding instead of the linear patch embedding approach. Their method had been divided into L stages that solved the affine registration with an image pyramid. All stages share the same architecture consisting of a convolutional patch embedding layer and Ni transformer encoder blocks. C2FViT solves the affine registration problem in a coarse-to-fine manner, and the intermediate input video Mi is transformed by progressive spatial transformation. Finally, the estimated affine matrix AL in the final stage is employed as the output of the model fθ. In this figure, L is three

Mok et al. proposed a unique application of transformer architecture to medical imaging [85]. In various medical imaging studies, rigid and affine registrations play a crucial role. The authors proposed a method named Coarse-to-Fine Vision Transformer (C2FViT) for 3D affine medical image registration (Fig. 9). Unlike traditional CNN-based techniques, this method demonstrated enhanced accuracy, robustness, and speed, particularly in scenarios with significant initial misalignment.

## Discussion

**Fig. 10:**
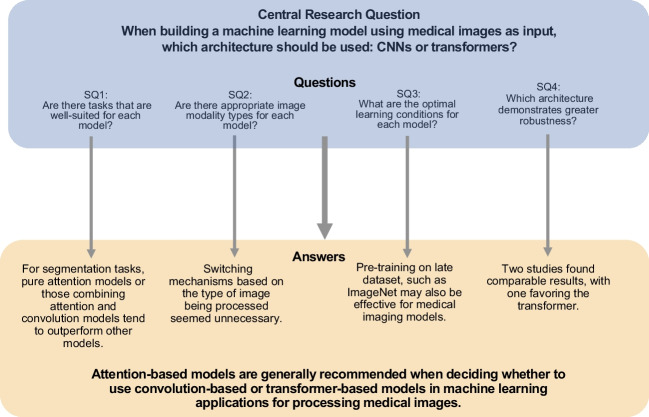
Visual summary of the central research question and key sub-questions addressed in the systematic review, along with their corresponding answers

Although we identified many studies that used attention, convolution, or a combination of both, few studies have directly compared these two approaches. Studies that do not simply cite results from other papers as benchmarks but instead run multiple models using the same dataset for comparison are particularly valuable. In addition, most studies rely solely on publicly available datasets, and few papers conducted comparisons using multiple datasets, including private datasets. As a result, it is difficult to fully answer our original research question and sub-questions. Nevertheless, we attempted to address these questions based on the available data. Figure 10 provides a visual summary of the central research question and SQs addressed in the systematic review, along with their corresponding answers.

### SQ1: Are There Tasks that are well Suited for each Model?

Although numerous studies suggest that attention mechanisms generally outperform convolution methods, our discussion focuses on studies that highlight scenarios where convolution is superior [65, 73, 75]. The summarized results are presented in Table 3. It is evident that tasks in which convolution excels are exclusively related to classification [65, 73, 75]. Given that there is only one example of registration, it is premature to draw definitive conclusions. In contrast, for segmentation tasks, pure attention models or those combining attention and convolution models tend to outperform other models. This superiority may stem from the ability of attention mechanisms to capture long-range dependencies and enable attention-based models to integrate information across the entire image [26, 97]. Consequently, incorporating attention mechanisms into the design of segmentation models may prove beneficial for achieving improved results.

**Table 3:** Studies highlighting instances where convolution proves to be superior

Title	Task category	Task	Dataset	Image type	Independent external test dataset	Pre-training	Best Architecture
Detecting tuberculosis-consistent findings in lateral chest X-rays using an ensemble of CNNs and vision transformers [65]	Classification	Tuberculosis or not	Detecting tuberculosis	X-ray	No	ImageNet	DenseNet-121
Investigating vision transformer models for low-resolution medical image recognition [73]	Classification	Specific classifications	DermaMNIST, BloodMNIST, PneumoniaMNIST, and OrganCMNIST	Low-resolution medical image	No	Unknown	CNN
On the effectiveness of 3D vision transformers for the prediction of prostate cancer aggressiveness [75]	Classification	Low-grade or high-grade	ProstateX-2	MRI	No	No (Scratch)	2D-CNN

### SQ2: Are There Appropriate Image Modality Types for each Model?

Upon examining datasets from studies asserting the superiority of convolution, no common characteristics emerged. Therefore, switching mechanisms based on the type of image being processed seemed unnecessary. However, Adjei-Mensah et al. noted that ViT models are susceptible to low-resolution medical images, suggesting that dataset quality could influence the choice of the mechanism [73].

### SQ3: What are the Optimal Learning Conditions for each Model?

The role of pre-training was noteworthy. Among the papers reporting superior or comparable transformer performances, approximately 59% (19 out of 32) described some form of pre-training. In contrast, only 25% (one out of four) of the papers favoring CNNs mentioned pre-training. Furthermore, ImageNet was frequently utilized for pre-training. Despite the belief that transformers benefit considerably from pre-training, this review indicates that pre-training on ImageNet may also be effective for medical imaging models.

### SQ4: Which Architecture Demonstrates Greater Robustness?

Regarding the most important SQ, when machine learning models are applied in real-world scenarios, it is unlikely that the learning and testing domains will be identical. A high degree of accuracy is required even when the two domains differ, underscoring the need for robustness. Only three studies used independent external test datasets to assess robustness [56, 67, 83]. Among these, two studies found comparable results, with one favoring the transformer. The similarity in results demonstrates the slight advantage of CNNs in terms of prediction accuracy. Appropriate model selection for each task can result in CNNs outperforming attention-based models in terms of robustness. However, this necessitates the availability of sufficient external test datasets in the selection process.

## **Central Research Question: When Building a Machine Learning Model Using Medical Images as Input**,** Which Architecture Should be Used: CNNs or Transformers?**

In conclusion, attention-based models are generally recommended when deciding whether to use convolution-based or transformer-based models in machine learning applications for processing medical images. This preference is based on various factors in addition to the above SQs. The first is the superior transparency of the attention-based models, which is critical for user confidence. Attention maps are excellent tools that can provide users with detailed insights. The second is the rapid development of foundation models that employ attention-based mechanisms, as exemplified by Meta’s SAM and MedSAM models [98, 99]. These models are anticipated to be central to future developments. Finally, addressing the question of whether mixed CNN and attention models should be adopted, our advice is against it unless there is a specific reason for adopting them. This recommendation is based on two primary reasons: first, complicating the model could impede the use of pre-trained models, which is critical for achieving high accuracy with attention-based models, and even if a successful model is developed, reusability for others could be challenging. Second, there is no evidence suggesting that mixed models are more robust.

### Limitations

This review is subject to certain limitations related to the chosen search terms. Given the relative novelty of attention-based models in the field, the available literature could potentially be skewed toward more favorable findings for these models. The newness of research on attention-based models might lead to an overrepresentation of optimistic results in the literature, as the scientific community tends to swiftly adopt promising new methods. Additionally, there is a potential for subjectivity in the selection of included studies. Despite our efforts to conduct a systematic and unbiased review, the inherent biases and interpretations of researchers may have influenced the results and conclusions drawn from the literature, and these factors cannot be completely eliminated. Referring to similar review articles in the field of general imaging could help mitigate this bias, ensure a more balanced perspective, and strengthen the validity of our conclusions.

## Conclusions and Future work

In this systematic review, we have comprehensively examined recent studies in medical image analysis to provide a detailed comparison of CNNs and attention-based models. Our analysis highlights that while both architectures have their distinct strengths, attention-based models hold great promise for advancing the field of medical imaging; however, it is important to recognize that attention-based models are relatively new in the field of medical imaging. This novelty means that their long-term performance and reliability have not yet been fully studied. Therefore, we emphasize the need for further research, particularly longitudinal studies, to determine the consistent effectiveness and potential limitations of attention-based models over time. In addition, future work should focus on refining these models, exploring hybrid architectures that combine the strengths of both CNNs and attention-based models, and evaluating their performance across a broader range of medical imaging tasks and modalities. By addressing these areas, the field can move closer to a more comprehensive understanding of the optimal use of these architectures, ultimately contributing to improved diagnostic accuracy and patient outcomes in clinical practice.

## Data Availability

No datasets were generated or analysed during the current study.
